# Counting on birth registration: mixed-methods research in two EN-BIRTH study hospitals in Tanzania

**DOI:** 10.1186/s12884-020-03357-1

**Published:** 2021-03-26

**Authors:** Sarah Reed, Josephine Shabani, Dorothy Boggs, Nahya Salim, Sillanoga Ng’unga, Louise T. Day, Kimberly Peven, Stefanie Kong, Harriet Ruysen, Debra Jackson, Donat Shamba, Joy E. Lawn, EN-BIRTH Study Group

**Affiliations:** 1 Maternal, Adolescent, Reproductive & Child Health (MARCH) Centre, London School of Hygiene & Tropical Medicine (LSHTM), London, WC1E 7HT UK; 2 Department of Health Systems, Impact Evaluation and Policy, Ifakara Health Institute (IHI), Dar es Salaam, Tanzania; 3 Department of Paediatrics and Child Health, Muhimbili Hospital University of Health and Allied Sciences (MUHAS), Dar Es Salaam, Tanzania; 4 Florence Nightingale Faculty of Nursing, Midwifery & Palliative Care, Kings College London, London, UK; 5 Implementation Research & Delivery Science Unit, Health Section, UNICEF, New York, NY USA; 6 School of Public Health, University of the Western Cape, Cape Town, South Africa

**Keywords:** Birth, Maternal, Newborn, Stillbirth, Survey, Civil registration, Birth certification, Vital statistics

## Abstract

**Background:**

Birth registration marks a child’s right to identity and is the first step to establishing citizenship and access to services. At the population level, birth registration data can inform effective programming and planning. In Tanzania, almost two-thirds of births are in health facilities, yet only 26% of children under 5 years have their births registered. Our mixed-methods research explores the gap between hospital birth and birth registration in Dar es Salaam, Tanzania.

**Methods:**

The study was conducted in the two Tanzanian hospital sites of the *Every Newborn*-Birth Indicators Research Tracking in Hospitals (EN-BIRTH) multi-country study (July 2017–2018). We described the business processes for birth notification and registration and collected quantitative data from women’s exit surveys after giving birth (*n* = 8038). We conducted in-depth interviews (*n* = 21) to identify barriers and enablers to birth registration among four groups of participants: women who recently gave birth, women waiting for a birth certificate at Temeke Hospital, hospital employees, and stakeholders involved in the national birth registration process. We synthesized findings to identify opportunities to improve birth registration.

**Results:**

Standard national birth registration procedures were followed at Muhimbili Hospital; families received birth notification and were advised to obtain a birth certificate from the Registration, Insolvency, and Trusteeship Agency (RITA) after 2 months, for a fee. A pilot programme to improve birth registration coverage included Temeke Hospital; hand-written birth certificates were issued free of charge on a return hospital visit after 42 days. Among 2500 women exit-surveyed at Muhimbili Hospital, 96.3% reported receiving a birth notification form and nearly half misunderstood this to be a birth certificate. Of the 5538 women interviewed at Temeke Hospital, 33.0% reported receiving any documentation confirming the birth of their child. In-depth interview respondents perceived birth registration to be important but considered both the standard and pilot processes in Tanzania complex, burdensome and costly to both families and health workers.

**Conclusion:**

Birth registration coverage in Tanzania could be improved by further streamlining between health facilities, where most babies are born, and the civil registry. Families and health workers need support to navigate processes to register every child.

## Key findings

**Table:** 

**What is known and what is new about this study?** • Nearly half of children in sub-Saharan Africa lack birth registration and thus have no legal “identity”. Linking rising facility births to birth registration could close the gap. • We explored the birth registration process in two of the EN-BIRTH study hospitals in Tanzania using mixed-methods. We interviewed > 8000 women after hospital birth and studied stakeholders’ perceptions through 21 in-depth interviews. We identified barriers and enablers to increasing birth registration following hospital birth and compared the standard national system to a pilot system. This is the first published study of its kind from Tanzania, and among the first in any low- and middle-income country setting. **What did we find and what does it mean?** • ***Exit survey findings***: At Muhimbili Hospital, using the standard birth registration process, 96.3% of women reported they had received a birth notification form, but nearly half confused it for a birth certificate. At Temeke Hospital, using the pilot initiative, 33.0% of women reported having received documentation to provide proof of the birth of their baby, and nearly all correctly reported this was not a birth certificate. • ***Qualitative research***: In-depth interview analysis showed birth registration is perceived as important, yet the process is complicated, confusing, under-resourced, and a burden on families and health workers. Women at both hospitals reported concerns regarding cost and time barriers to travel to the registration office or hospital to obtain a birth certificate. Hospital staff at Muhimbili reported on the constraints and inefficiencies of a paper-based system. Health workers in the pilot scheme at Temeke Hospital reported resource constraints, a lack of training opportunities and no formal job descriptions to support birth registration related duties. National stakeholders were concerned with the pilot birth registration scheme efforts regarding sustainability and the official acceptability of hand-written certificates. **What next and research gaps?** The birth registration process in Tanzania requires further simplification. Investment is needed to reduce context-specific barriers for health workers and families, including burden of time and cost. Innovation and implementation research should target solutions to close the gap between facility-births and birth registration, addressing these many missed opportunities for every newborn to be counted.	

## Background

Birth registration is the process by which the event and characteristics of a child’s birth are recorded in a country’s civil registry [1]. At an individual level, proof of registration, usually in the form of a birth certificate, is essential to accessing citizenship in terms of basic rights and services including education, health care, land ownership, and formal employment [2, 3]. Sustainable Development Goal (SDG) target 16.9 specifically aims to provide legal identity for all, including birth registration, by 2030 [4].

At the population level, birth registration in the Civil Registration and Vital Statistics (CRVS) system provides continuous demographic information for programming and planning [5, 6]. Comprehensive CRVS could enable more timely population data to track progress towards the SDG aspiration to “leave no-one behind”.

Despite its importance, current estimates suggest that nearly a quarter of the world’s children are unregistered [9]. Sub-Saharan Africa has the lowest coverage, with fewer than half of children under 5 years registered [10]. Commonly reported barriers in low- and middle-income countries (LMICs) include lack of awareness of the importance of birth registration, cost, and travel distance to registry offices [11–15].

Since facility births are increasing in all regions of the world, new opportunities exist to improve birth registration [16, 17]. Typically, families are given a birth notification after facility birth and instructed how to obtain the formal birth certificate at the civil registry. Many countries are now simplifying this process by direct linkage with health facilities, including giving health workers responsibility for birth registration, or automatic electronic registration at the time of notification [18].

In Tanzania, large gaps remain between facility births and birth registration. Nearly two-thirds of births are now in a health facility, and yet only 25% of children under 5 years are registered – one of the lowest rates worldwide [2, 19]. No research has been published focusing on understanding the barriers to low birth registration after facility birth and opportunities to close this gap.

Our study in Tanzania was nested within two of the five hospitals in the *Every Newborn* – Birth Indicators Research Tracking in Hospitals (EN-BIRTH) study. The *Every Newborn* Action Plan (ENAP), agreed by all United Nations member states, aims to end preventable newborn deaths and stillbirths [7]. The linked measurement improvement roadmap prioritised counting every newborn through both birth and death registration, and underlined the need for innovation in routine facility data to better monitor progress and target inequalities [8]. As part of the *Every Newborn* measurement improvement roadmap, EN-BIRTH was an observational study with mixed-methods in three countries (Tanzania, Bangladesh and Nepal) that aimed to validate the measurement of selected newborn and maternal indicators for routine facility-based tracking of coverage, quality of care, and outcomes [8, 20].

## Objectives

This paper is part of a supplement based on the EN-BIRTH multi-country validation study, ‘*Informing measurement of coverage and quality of maternal and newborn care*’ and focuses on birth registration with three objectives:

1. **Describe BIRTH NOTIFICATION AND REGISTRATION PRACTICES** in two hospitals in Dar es Salaam, Tanzania.
2. **Analyse WOMEN'S REPORT of birth notification and registration processes** at exit interview survey after hospital birth.
3. **Evaluate perceived BARRIERS AND ENABLERS to birth registration** in Dar es Salaam, Tanzania from women, hospital employees and key national birth registration stakeholders.

## Methods

### Settings

The EN-BIRTH study hospitals in Tanzania both provide Comprehensive Emergency Obstetric and Neonatal Care (CEmONC). Muhimbili National Hospital is an urban referral and teaching hospital in central Dar es Salaam and follows the current national process for birth registration. Temeke Regional Hospital is in the southern district of Dar es Salaam and is a site for the new, free birth registration pilot program (Additional file 1).

### Study design and analyses according to objective

This birth registration study uses primary data collected during the EN-BIRTH study and the detailed study research protocol and validation results have been published separately [20, 21].

### Objective 1: Practices of birth notification and registration after birth in two hospitals in Dar es Salaam, Tanzania

The first author and a research assistant from Ifakara Health Institute (IHI) whilst observing the birth registration practices informally interviewed 3–4 staff involved in the process at each hospital for further clarification. We identified critical staff to be formally interviewed for Objective 3.

### Objective 2: Women’s exit survey report of birth registration process

Trained EN-BIRTH study data collectors exit-interviewed consenting women immediately after discharge from the postnatal wards (July 2017–2018) [20]. Eligible women had been observed with consent during hospital birth and had a live child on discharge. We asked closed and open-ended questions pertaining to the birth registration process: 1) whether birth notification was received, 2) whether birth certificate was received, 3) whether they knew how to obtain a birth certificate, 4) when they planned to register the birth and 5) any process concerns (Additional file 2). Interviews were conducted in Swahili and free-text answers were translated into English prior to analysis. We calculated gaps in reported coverage of receiving a birth notification/certificate, and stratified by women’s characteristics, including education level, and by wealth quintiles calculated by principal component analysis.

Background characteristics analyses were undertaken using Stata Version 16 (StataCorp, 2019 College Station, TX) and other analyses and figures were generated using R statistical programming software (version 3.6.3) [22, 23].

### Objective 3: Barriers and enablers to birth registration

We recruited four groups of consenting participants who interacted with the facility-based birth registration process to capture a variety of views: a) women on the postnatal or the kangaroo mother care (KMC) wards at both hospitals (Muhimbili *n* = 4, Temeke *n* = 3) b) women who had returned to the hospital to obtain a birth certificate at Temeke (*n* = 3) c) hospital employees involved in the birth registration process at both hospitals (Muhimbili *n* = 3, Temeke *n* = 4) d) key stakeholders involved in national-level birth registration efforts (*n* = 4). We purposively sampled for groups a) b) and c). We snowball sampled group d), which was initiated by a key informant at the Ministry of Health, and included two Ministry of Health officials, an officer at the Registration, Insolvency, and Trusteeship Agency (RITA) and a United Nations Children's Fund (UNICEF) employee involved in the birth registration pilot program at Temeke hospital.

Interview guides on barriers and enablers to birth notification and registration practices were drafted by the first author after review of the literature and in collaboration with EN-BIRTH study co-authors. The guides were translated into Swahili and revised for local acceptability after pilot testing (Additional file 3). The experienced qualitative research assistant from IHI and the first author conducted semi-structured in-depth interviews in English or Swahili, as preferred by the participants, whilst ensuring privacy on the ward or office. The sample size was determined when both interviewers agreed saturation had been reached. Interviews conducted in Swahili were translated verbatim in real-time into English by the research assistant. All interviews were recorded, transcribed, translated, anonymized and stored on a secure server.

The first author completed an inductive thematic content analysis to identify key perceived barriers and enablers to birth registration [24]. NVivo10 software was used for coding and data management. The first author explored the transcripts through multiple readings for general impression and generated initial codes inductively for emerging themes. To improve the trustworthiness of the results, co-authors contributed to grouping of codes with similar concepts into sub-themes/themes for interpretation. Consensus was obtained regarding any difference in interpretation. Respondent groups were coded prior to triangulation, then relationships between the groups were examined (Additional file 4). Reliability and credibility of findings were attained through a prolonged research engagement with Tanzanian co-authors and interpretation of results between researchers and through communication with hospital staff. Detailed records were maintained throughout data collection and analysis to strengthen dependability of results.

## Results

### Objective 1: Practices of birth notification and registration

#### Tanzanian national policy and process

**Fig. 1:**
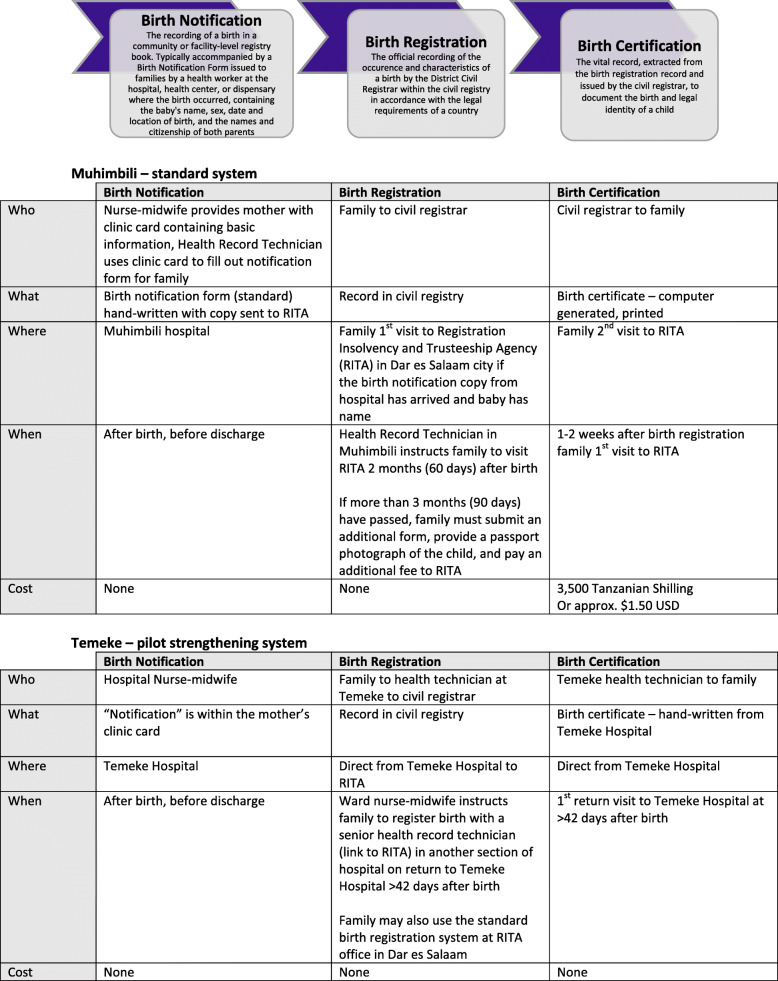
The process of birth registration in two health facilities in Tanzania, EN-BIRTH study

RITA oversees the legal requirement of birth registration in Tanzania within 90 days of birth (Fig. 1). The names of the child and both parents must be provided in order to receive a birth certificate. The process involves parents or an occupant of the child’s household visiting the District Administrative Office twice. The first visit is to submit the “Notification of Birth” form received from any facility birth, request the birth certificate, and pay the 3500 Tanzanian Shillings ($1.50 USD) fee. The second visit is 1–2 weeks later for collection of the printed birth certificate. Registrations more than 90 days after birth are considered “late”, and additional fees and procedures apply [25–27].

#### Muhimbili National Hospital – standard Tanzania birth registration system

Observations and interviews at Muhimbili revealed that typically, nurse-midwives assigned all women who delivered at the hospital a delivery number and issued each a clinic card containing basic information including the time of delivery and sex of the baby at birth. Within 24 h, a health record technician visited the postnatal ward and, using information from the clinic card, issued the birth notification to the mother before her discharge. The notification form was a hand-written, detachable form from a pre-printed book leaving a carbon-copy for delivery to the RITA office monthly. The health technician also informed the women of the importance of birth certification (e.g. school admission, student loans, national identification card, future employment, and international travel) and explained the process: after 2 months to take the birth notification form to the RITA office in Dar es Salaam to purchase the printed birth certificate. This advice specifically incorporated an extra 30-day window before they would begin to incur “late registration” fees.

#### Temeke Regional Hospital – pilot facility-linked birth registration system

According to health workers at Temeke, in 2012, the government of Tanzania, with the support of UNICEF and funding from the Canadian Government, launched a pilot parallel birth registration system at select community-level facilities to provide free, hand-written birth certificates. Temeke Hospital was one of these selected pilot facilities where the nurse-midwife issued a delivery card containing birth information to the mother, instead of a birth notification card. Prior to discharge, the nurse-midwives gave instructions to women in groups, typically within 6 h of birth, with information on how to register their baby’s birth. Women were advised they could receive one free hand-written birth certificate on presentation of both their delivery card and their child’s clinic card at Temeke Hospital 42 days after birth. The birth certificates are generated from an official RITA form. The lower portion is the birth certificate and is given to the parent. The top portion is sent to RITA containing the child’s name, family information, date and place of birth. The timeframe of returning after 42 days is based upon the custom of naming a child at 40 days, due to the belief that until then, the baby has not surpassed the vulnerable early stages of survival. The certificate provided on the return visit takes approximately 2 h to issue after arriving at the facility. In addition, birth registration information was to be sent directly to RITA by SMS using a designated mobile phone, however, this was not observed during the time of the study due to reported issues with charging the battery of the device.

The existing systems in both hospitals were entirely paper based.

### Objective 2: Women’s exit survey report of birth registration process

**Fig. 2:**
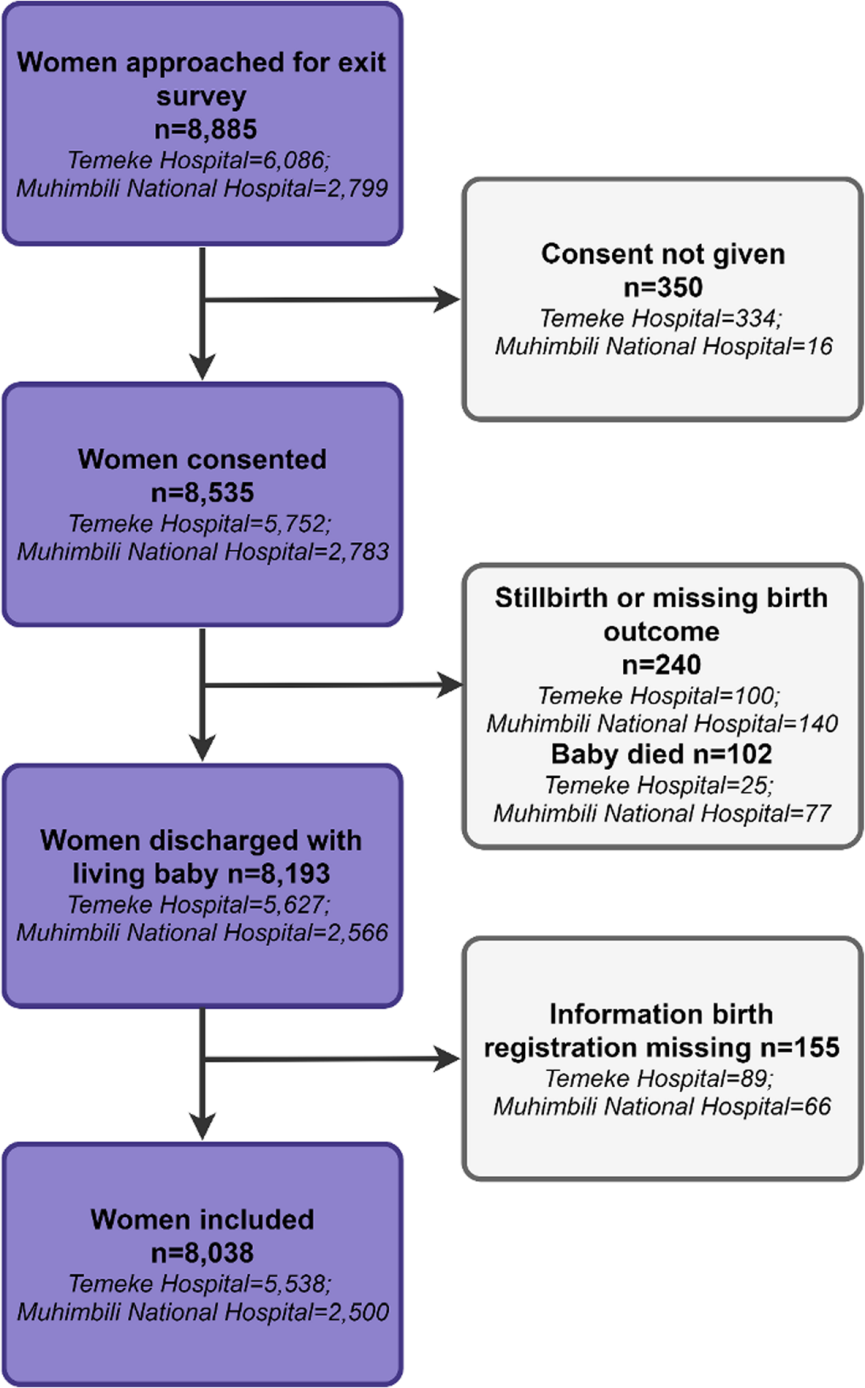
Flow diagram for birth registration exit survey at Muhimbili and Temeke, EN-BIRTH study (*n* = 8038)

Among 8885 women approached for exit survey, 8535 consented and 8193 were discharged with a live baby and eligible for questions on birth registration (Fig. 2). 8038 (5538 in Temeke, 2500 in Muhimbili) were included in the analysis after missing data were excluded. Background characteristics of women and newborns included in the analysis are shown in Table 1. Most women had some secondary education or higher (62.8% in Muhimbili and 37.3% in Temeke). While 59.8% of women had caesarean births in Muhimbili, 5.5% had caesarean births in Temeke.

**Table 1:** Characteristics of respondents at Muhimbili and Temeke Hospitals, EN-BIRTH study (*n* = 8038)

	Temeke Regional Hospital n (%)	Muhimbili National Hospital n (%)
**Total**	**5538**	**2500**
**Maternal age**		
< 18 years	15 (0.3)	4 (0.2)
18–19 years	618 (11.2)	113 (4.5)
20–24 years	1900 (34.3)	498 (19.9)
25–29 years	1408 (25.4)	798 (31.9)
30–34 years	955 (17.2)	654 (26.2)
35+ years	642 (11.6)	433 (17.3)
**Maternal education**		
No education	163 (2.9)	47 (1.9)
Primary incomplete	106 (1.9)	40 (1.6)
Primary complete	3178 (57.4)	834 (33.4)
Secondary incomplete	1242 (22.4)	789 (31.6)
Secondary complete or higher	826 (14.9)	781 (31.2)
Don’t know	23 (0.4)	9 (0.4)
**Parity**		
Nullipara	2331 (42.1)	932 (37.3)
Multipara	3197 (57.7)	1565 (62.6)
Don’t know	9 (0.2)	2 (0.1)
**Mode of birth**		
Vaginal births	5231 (94.5)	1005 (40.2)
Caesarean section	307 (5.5)	1495 (59.8)

**Fig. 3:**
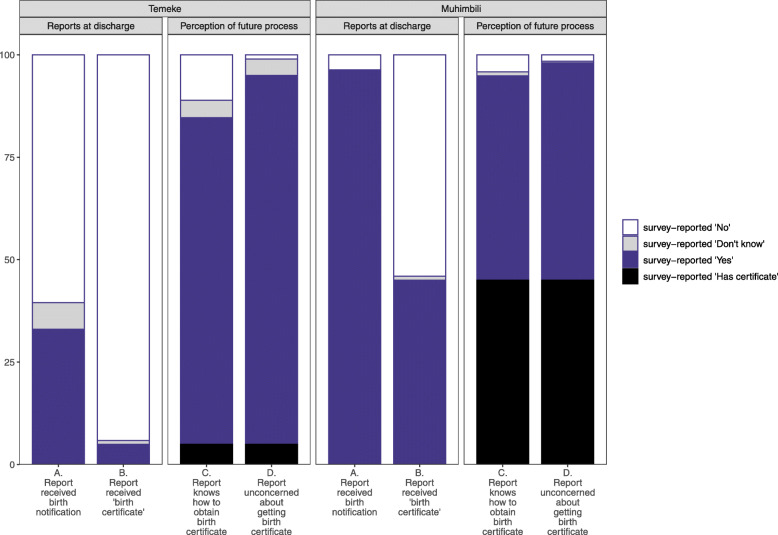
Gaps in birth notification and certificate coverage, EN-BIRTH study (Muhimbili *n* = 2500, Temeke *n* = 5538)

Among 2500 women interviewed in the exit survey at Muhimbili Hospital, 96.3% responded they had received a birth notification, < 1% responded that they didn’t know or remember (Table 2, Fig. 3). 45.0% of women reported having received a birth certificate.

**Table 2:** Women reporting receiving notification or birth certificate, EN-BIRTH study (*n* = 8038)

	Temeke Regional Hospital	Muhimbili National Hospital
	n (%)	n (%)
**Total mothers with living babies**	5538 (100)	2500 (100)
Receive any birth notification form or other relevant documentation to provide proof of birth of baby		
Yes	1828 (33)	2407 (96.3)
No	3348 (60.5)	92 (3.7)
Don’t know	362 (6.5)	1 (0)
Baby received any birth certificate		
Yes	274 (4.9)	1,125 (45)
No	5213 (94.1)	1351 (54)
Don’t know	51 (0.9)	24 (1)
**Total mothers with living babies not yet receiving certificate**	5264 (100)	1375 (100)
Knowledge of how to obtain birth certificate for the baby		
Yes	4415 (83.9)	1247 (90.7)
No	614 (11.7)	104 (7.6)
Don’t know	235 (4.5)	24 (1.7)
Timing of planning to get the birth certificate		
Within 30 days	5 (0.1)	94 (6.8)
Within 30–60 days	4 (0.1)	1060 (77.1)
More than 60 days	4405 (83.7)	94 (6.8)
Don’t know	850 (16.1)	127 (9.2)
Had any concerns about getting a birth certificate		
Yes	57 (1.1)	39 (2.8)
No	4984 (94.7)	1326 (96.4)
Don’t know	223 (4.2)	10 (0.7)

At Temeke Hospital, among 5538 women interviewed 33.0% reported receiving a birth notification or similar and 6.5% responded “Don’t know/remember”. 4.9% of women reported receiving a birth certificate, and 0.9% reported that they didn’t know or remember.

There were no substantial differences in responses amongst women of different education levels. “Birth notification not received” response stratified by wealth quintile was reported among 31.5% of the lowest quintile compared to 15.2% highest quintile in Muhimbili, and “don’t know” responses in Temeke by 32.6% in the lowest compared to 13.3% in the highest. Among respondents correctly reporting no birth certificate had been received at discharge, 17.7% were from the lowest and 27.1% from the highest quintile in Muhimbili but more similar in Temeke (19.8 to 19.7%) (Additional file 5).

**Fig. 4:**
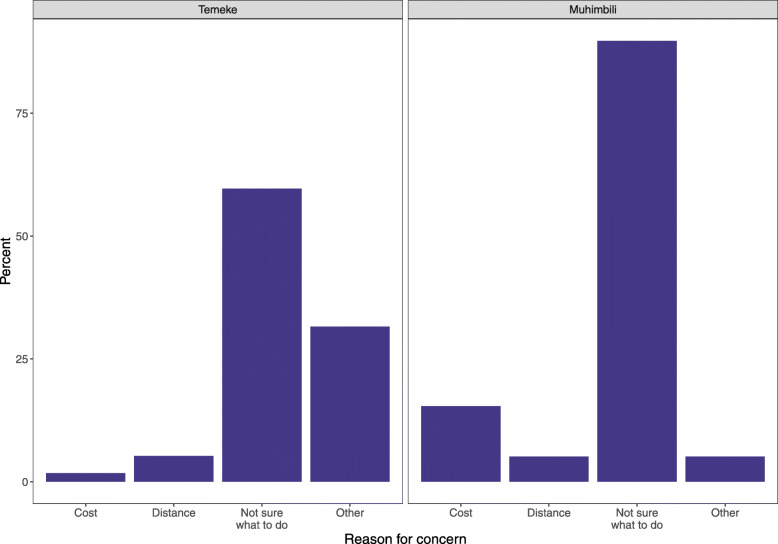
EN-BIRTH study survey: “What are your concerns [about getting a birth certificate]?” Muhimbili (*N* = 39), Temeke (*N* = 57). Respondents were permitted to select multiple choices

Among women who reported they had not yet received a birth certificate, 90.7% at Muhimbili and 83.9% at Temeke Hospital reported they knew how to obtain one. Three-quarters (77.1%) of women in Muhimbili planned to get the birth certificate in 30–60 days while 83.7% of women in Temeke planned to wait more than 60 days. Only 2.8% of respondents at Muhimbili Hospital and 1.1% at Temeke Hospital expressed concerns with obtaining a birth certificate. Cited reasons include: not knowing what to do or not informed/educated (*n* = 77), cost (*n* = 7), distance (*n* = 5), bureaucracy (*n* = 1) and not having received a birth notification (*n* = 1) (Fig. 4).

### Objective 3: Barriers and enablers to birth registration

A total of 21 in-depth interviews with key informants were completed. Interviews lasted an average of 21 min in duration and no repeat interviews were conducted.

#### Muhimbili Hospital

Women generally understood the importance of birth certification, and often provided specific reasons for their motivation to obtain a birth certificate for their child. One woman specifically cited:

*“The good thing is [the birth certificate] reminds about the date of birth of the baby, also it helps in school admission even in the future when she wants to get a job it can be useful.”* -Woman, Muhimbili Hospital

Every respondent stated they did not understand or remember some aspect of the instructions about the process e.g., where and when to go, how much it would cost, and the distance to RITA. Given Muhimbili is a referral hospital, it is not uncommon for women to travel a long distance to give birth at the hospital, so the need to return to downtown Dar es Salaam twice to register their child at RITA was a concern:

*“I am living at Bagamoyo, so there is a distance. And to tell you the truth, from Bagamoyo to the office which provides the birth certificates is too far.”* -Woman, Muhimbili Hospital

Another woman shared her concern that she might lose the birth notification issued to her during the two-month period she was instructed to wait before registration, and a hospital staff also explained:

“*The challenge is ….according to the police, we can’t reissue the notification.”* -Health worker, Muhimbili Hospital

Health workers expressed general satisfaction with the current system, especially with the designated health technician who issues birth notification cards and instructions. The main identified barrier under this process was the inefficiency of a paper-based system. Hospital staff reported the hand-written birth notification system increased burden on staff, potential for error, and difficulty retrieving files. Once the notification form book is delivered to RITA, it cannot be corrected, and copies cannot be given to families who have lost their form. To address these issues, a staff member interviewed recommended:

*“Improving the [electronic medical record] system so that all records are available in the computer system to avoid paperwork which is difficult to keep.”* -Hospital staff, Muhimbili Hospital

#### Temeke District Hospital

All women interviewed expressed the importance of possessing a birth certificate as a prerequisite for access to school, travel, and work, and approved of the birth certificates being free of charge and provided on the same day.

The most consistently reported barrier was incomplete or confusing information on the process: “unclear and inaccurate information on how and where to get the birth certificates”, particularly around the issue of “the right date to get the certificate”. Some women understood after 42 days, others mentioned 40 or 43 days, and several did not remember. Despite the effort to improve access by bringing the new birth certification process to Temeke Hospital, distance was still an issue:

*“Sometimes [the process] is difficult to follow because there are people who are living far away from here. It may happen that they forgot to come with two cards, so they will be forced to go back to where they are staying to get them because here you cannot receive the birth certificate without those two cards. Since they are staying far away from here, they will be forced to come again on another day.”* -Woman, Temeke Hospital

One woman expressed an important concern over the nature of the hand-written birth certificates being issued, explaining that the certificates issued at Temeke Hospital are not permanent. To address this, she suggested that Temeke Hospital provide permanent certificates, similar to those issued by RITA, to prevent future inconvenience.

Health workers supported the concept of issuing birth certificates free of charge in a district/regional hospital and noted an increased awareness regarding birth certification since the introduction of the new programme. However, several barriers were still reported. First, they described being understaffed and overstretched by the administrative burden of completing the delivery cards and logbooks in addition to responding to a high volume of births. Staff shortage was specifically cited by the health worker in charge of hand-writing birth certificates – when she has a day off, there is no one to cover the work. The second barrier expressed was the need for additional training and support. The new birth registration responsibilities were not included in the job descriptions of health workers responsible for implementing them. One nurse expressed that additional support and training opportunities would improve her motivation, but they are currently only offered to managers.

Finally, a lack of resources was expressed by several health workers. Although women are supposed to stay for 24 h after birth, they are forced to discharge more quickly, typically after only 6 h due to high volume of births and limited capacity. Health workers must then provide women with the documentation and instructions within a shorter time, while responding to other duties, and women, soon after birth, are expected to retain these documents and information. Since there was no designated physical space for birth certification services, the nurse issuing certificates expressed difficulty in staying organized. The child’s clinic cards are required as proof of birth, however, occasionally the hospital runs out of cards to issue.

### National stakeholders involved in birth registration process

Stakeholders reported that the existing birth registration system is highly centralized with a shortage of staff and resources to support timely registration. Several stakeholders cited inadequate funding as a source of constraint for implementing sustainable programs and discussed the system’s current dependence on donor organizations.

*“[UNICEF’s hand-written certificate initiative] is not yet owned by the government. You know, we depend on some sort of fund- that’s where the problem is.”* -Ministry official

Another issue with the lack of full government ownership of the birth registration process piloted at Temeke Hospital is that the handwritten birth certificates may not always be recognized as official. Several stakeholders suggested that families would still eventually need to purchase a printed certificate for official use. Another respondent shared that Tanzania planned to scale-up the new system at the administrative ward-level, potentially increasing government ownership, although there were no known plans to replace the existing system in larger hospitals such as Muhimbili.

## Discussion

Our study identifies current barriers and enabling opportunities to improve birth registration following hospital birth in Tanzania. We compared the standard registration system with a pilot improvement scheme – the first published study from Tanzania, and among the first in any LMIC setting.

We found that women who gave birth in hospital highly value the importance of birth registration, expressing an earnest desire for their child to have a birth certificate and providing examples of how it would benefit their wellbeing. Yet families are often blamed for failure to register births without adequate information and acknowledgement of their intentions [28]. Their stated intention to obtain the certificate does not correlate with the large facility birth registration gap in Tanzania, suggesting more focus needs to be placed on understanding and addressing the real barriers families face [29].

We found the complex processes of birth notification, registration and certification were the major barriers preventing families from fulfilling their intentions. Respondents were confused by the multi-step process and strikingly nearly half of respondents from Muhimbili reported having received a birth certificate at discharge, which was not currently possible. This alone could be a major contributor to why some families never take the next step to visit the civil registry if they misunderstand the birth notification form received from the hospital as a birth certificate. By contrast, under the pilot scheme in Temeke, 94% of women were clear they had not yet received a birth certificate at discharge, although were unsure about other aspects of process, including when to return and which documents were required.

Instructions about the birth registration process in both hospitals are directed at women during the early postpartum period who are typically discharged within 6–24 h of giving birth. During this early postnatal period, women are busy recovering, caring for their newborn baby, preparing to travel home and receiving other information from their health providers. Providing written and pictorial instructions for women to take home could help navigate these complexities. Including the wider family and especially the baby’s father or other primary caregivers in hearing these birth registration instructions would also provide opportunities for clarifying questions to be addressed. Antenatal care visits provide additional opportunity to discuss the process and share information before birth, as over half of women in Tanzania attend four or more antenatal visits [18, 19, 29].

The travel distances twice to the RITA office in Dar es Salaam to obtain the certificate were reported as significant barriers to birth registration. This concern was still expressed for the pilot scheme in Temeke Hospital despite the advantage of only one visit and potential to combine with attending for a postnatal check.

Cost of the birth certificates was cited as a barrier by women at Muhimbili Hospital, in addition to opportunity costs of having to travel to the RITA office twice. Although Temeke Hospital was piloting free certificates and only one visit was needed, opportunity costs were still highlighted in terms of travel and waiting time. Women interviewed in Temeke with experience of the standard registration process with their previous children reported preference for the pilot process. Since 70% of the Tanzanian population live on less than $2 USD per day, all financial and opportunity costs associated with birth registration increase hardship for the poorest [30]. This is likely to increase the equity gap, reducing birth registration for the poorest, or those living rurally, distant from RITA offices.

Overcoming barriers to birth registration goes beyond family considerations. Health workers involved in the birth notification/registration process at both facilities shared their concerns about their high clinical workloads, shortage of staff, and how birth notification and registration was adding to their general administrative burden. Health workers had difficulty filling out the required paperwork while also trying to provide care. These challenges crosscut with the inefficient paper-based records system, and the difficulty of replacing lost birth notifications or clinic cards. This was more pronounced by health workers at Temeke Hospital with additional responsibility for issuing handwritten birth certificates. The lack of consistent physical facility space for birth registration, no colleague cross-cover for absence or leave, and job descriptions not updated to include these new responsibilities were stated. Many staff expressed the need for birth registration-specific training, sharing that current training programs are usually reserved for managers, and not for implementing staff.

### Strengths and limitations

The strengths of our study include the mixed-method design using a large survey sample (> 8000 women) in addition to qualitative methods. We compared two different processes currently in use in Tanzanian hospitals. Our results detail not only the registration processes but include the experiences of both women and health workers, which are currently underreported in existing literature.

However, our study also has limitations. Both hospitals are peri-urban/urban referral facilities within the limits of the country’s most populous region, which may affect generalizability of results. Our sample of women has a higher education level than average in the Tanzanian population, which may account for lack of variation in women’s response by educational level [19]. Studies in other settings have found that registration rates tend to be higher among women with higher levels of education [8, 10, 17]. We anticipate the challenges for women may be even greater for those in rural areas, and after home births. Therefore our results will have relevance for other hospitals in Tanzania or other countries with similar birth registration processes.

The purposive sampling and timing of the qualitative interviews with women must also be taken into consideration. All women interviewed on the postnatal wards had given birth mostly within 24 h and thus may have very recently received information on the birth registration process.

The sample size for the in-depth interviews was relatively small yet many concerns were identified, which notably were not captured by the closed-question exit survey despite much larger sample size. Although data saturation was determined to be reached, the study may have benefited from a larger sample size for further comparison between hospitals.

An important gap in our study is that we did not capture whether birth notifications had been provided to the 342 (4%) of women who had experienced stillbirth or neonatal death nor their understanding regarding death registration. This was to avoid further distress immediately after the loss of a child. Worldwide about 4% of neonatal deaths have a death certificate [31]. A multi-country study (five demographic surveillance sites in Africa and Asia) tested questions for use in household surveys regarding birth and death registration and found only 1.2% of neonatal deaths and 2.5% of stillbirths were reported to be registered, despite high facility birth rates [32].

### Programmatic implications and research gaps to close the facility birth registration gap

In the past, attention has been focused on closing the gap between high immunization coverage and low birth registration coverage. Recent UNICEF reports suggest that similar methods are applicable to closing the gap between high facility birth and low birth registration [2, 6, 33]. For the Tanzanian settings, our study findings support strategies suggested from other settings, including strengthening registration processes within or close to hospitals and supporting health workers. Other possibilities that we did not examine include creating awareness during antenatal visits, large-scale public health campaigns and multi-channel messaging on the importance of registration and how to get registered [34].

To improve coverage of birth registration, current systems require reducing the burden for both families and health workers. The existing system needs additional simplification and greater accessibility for families. Designated staff also need to be motivated by having registration-related duties explicitly stated in their job descriptions and by receiving training and supportive supervision.

Whilst improving CRVS systems at the facility level, there are opportunities to ensure these data are linked with routine Health Management Information Systems (HMIS). Digitisation may facilitate interoperability between information systems for data usage [8]. Improving digital systems in facilities may reduce the burden on staff to document multiple data sources. Digitization could also enable data to flow between facilities and district offices more easily, allowing registration to occur in the child’s local district, regardless of where they were born, reducing burden on families navigating a multi-step process that the existing system creates. However, digitization itself also has potential challenges in introduction and in sustainability [35, 36].

## Conclusions

Birth registration systems in many parts of the world remain highly centralized, cumbersome and costly for both providers and families. Tanzania has strengthened its birth certification process with pilot schemes linked to increasing facility births. Many missed opportunities exist to further lift the burden of responsibility from families and health workers by simplifying the process and testing further innovations, such as giving the birth certificate at the time of discharge or through electronic registration. Context-specific adaptations with evaluation are needed to inform the most effective strategies for each country, including Tanzania, and accelerate progress for birth registration coverage so that every newborn everywhere counts.

## Supplementary Information

**Additional file 1.** EN-BIRTH study sites – National mortality rates and hospital context.

**Additional file 2.** EN-BIRTH study exit survey questions.

**Additional file 3.** Birth registration in-depth interview guides, EN-BIRTH study.

**Additional file 4.** Birth registration thematic content analysis, EN-BIRTH study.

**Additional file 5.** Exit survey-reported birth notification and certificate coverage, stratified by education level and socioeconomic status.

**Additional file 6.** Ethical approval of local institutional review boards, EN-BIRTH study.

## Data Availability

The datasets generated during and/or analysed during the current study are available on LSHTM Data Compass repository, https://datacompass.lshtm.ac.uk/955/.

## Abbreviations

CEmONC: Comprehensive Emergency Obstetric and Neonatal Care
CRVS: Civil Registration and Vital Statistics
ENAP: *Every Newborn* Action Plan now branded as *Every Newborn*
EN-BIRTH: *Every Newborn*-Birth Indicators Research Tracking in Hospitals study
HMIS: Health Management Information Systems
IHI: Ifakara Health Institute
KMC: Kangaroo Mother Care
LMIC: low- and middle-income countries
LSHTM: London School of Hygiene & Tropical Medicine
MARCH Centre: Maternal, Adolescent, Reproductive & Child Health Centre, LSHTM
RITA: Registration, Insolvency, and Trusteeship Agency in Tanzania
SDG: Sustainable Development Goal
UNICEF: United Nations Children’s Fund
