# Supplementary material for: Counting on birth registration: mixed-methods research in two EN-BIRTH study hospitals in Tanzania
Source: BMC Pregnancy Childbirth. 2021 Mar 26;21(Suppl 1):236. doi: 10.1186/s12884-020-03357-1 (PMC7995691; doi:10.1186/s12884-020-03357-1)
Supplement: Supplementary file 1 — Additional file 1. EN-BIRTH study sites – National mortality rates and hospital context. [file 12884_2020_3357_MOESM1_ESM.pdf]

Every Newborn BIRTH multi-country validation study: informing measurement of coverage and quality of maternal and newborn care

Counting on birth registration: mixed-methods research in two EN-BIRTH study hospitals in Tanzania

Additional File 1: EN-BIRTH study sites – National mortality rates and hospital context

**Country Context**	**Tanzania**	
**National mortality rates at start of EN-BIRTH study (2016)**		
MMR/ 100,000 live births[1]	398	
NMR/ 1000 live births[2]	22	
SBR/ 1000 total births[3]	22	
% Institutional Births (2016)[4]	62.6	
**EN-BIRTH Study Hospitals**		
**Name**	Temeke Regional Hospital, Dar es Salaam	Muhimbili National Hospital, Dar es Salaam
**Hospital type**	Regional	National
**Total births annual 2017-18**	11,609	8,233

References:

1. WHO, UNICEF, UNFPA, World Bank Group, and the United Nations Population Division: Trends in maternal mortality: 1990 to 2015. In. Geneva 2015.

2. UNICEF, WHO, World Bank, UN-DESA Population Division: Levels and trends in child mortality 2015. In. New York 2015.

3. Blencowe H, Cousens S, Jassir FB, Say L, Chou D, Mathers C, Hogan D, Shiekh S, Qureshi ZU, You D: National, regional, and worldwide estimates of stillbirth rates in 2015, with trends from 2000: a systematic analysis. The Lancet Global Health 2016, 4(2):e98-e108.

4. World Health Organization: Global Health Observatory data repository.
