# Supplementary material for: Counting on birth registration: mixed-methods research in two EN-BIRTH study hospitals in Tanzania
Source: BMC Pregnancy Childbirth. 2021 Mar 26;21(Suppl 1):236. doi: 10.1186/s12884-020-03357-1 (PMC7995691; doi:10.1186/s12884-020-03357-1)
Supplement: Supplementary file 2 — Additional file 2. EN-BIRTH study exit survey questions. [file 12884_2020_3357_MOESM2_ESM.pdf]

Every Newborn BIRTH multi-country validation study: informing measurement of coverage and quality of maternal and newborn care

Counting on birth registration: mixed-methods research in two EN-BIRTH study hospitals in Tanzania

Additional File 2: EN-BIRTH study exit survey questions

Question #	Tablet prompt	Tablet answer options	Next questions
36	Did you receive a birth notification form or other relevant documentation to provide proof of the birth for your baby?<br>SHOW FORM	Yes	Go to question 37
		No	Go to question 37
		Not applicable baby died	**skip** to next section (question 41 onwards)
		Don't know/don't remember	Go to question 37
37	In addition to this notification, a baby should also receive birth certification. Did you receive this form?<br>SHOW FORM	Yes	**skip** to next section (question 41 onwards)
		No	Go to question 38
		Don't know/don't remember	Go to question 38
38	Do you know how to obtain such a birth certificate for your baby?<br>NOTE: provide birth certificate information leaflet afterwards	Yes	Go to question 39
		No	Go to question 39
		Don't know/don't remember	Go to question 39
39	When are you planning to get your birth certificate? DEPENDING ON MOTHER'S ANSWER, WRITE ANSWER IN EITHER WEEKS OR MONTHS	[Free text number]	Go to question 40
	Weeks/Month	[Select one of]<br>Weeks	
		Months	
		Don't know/don't remember	
40	Do you have any concerns about getting a birth certificate?	Yes	Go to question 40a
		No	Go to next section (question 41 onwards)
		Don't know/don't remember	Go to next section (question 41 onwards)
40a	If Yes, what are your concerns? MULTIPLE SELECTION POSSIBLE-SELECT ALL RELEVANT ANSWER OPTIONS	[Select yes/no] Distance/too far –	Go to next section (question 41 onwards)
		Cost	
		Not sure what to do –	

		Other-	
		Other, specify ___[Free text]___	
		Don’t know/don’t remember	
Interviewer Comments		___[Free text]_____	
