# Supplementary material for: Counting on birth registration: mixed-methods research in two EN-BIRTH study hospitals in Tanzania
Source: BMC Pregnancy Childbirth. 2021 Mar 26;21(Suppl 1):236. doi: 10.1186/s12884-020-03357-1 (PMC7995691; doi:10.1186/s12884-020-03357-1)
Supplement: Supplementary file 3 — Additional file 3. Birth registration in-depth interview guides, EN-BIRTH study. [file 12884_2020_3357_MOESM3_ESM.pdf]

Every Newborn BIRTH multi-country validation study: informing measurement of coverage and quality of maternal and newborn care

Counting on birth registration: mixed-methods research in two EN-BIRTH study hospitals in Tanzania

Additional File 3: Birth registration in-depth interview guides, EN-BIRTH study

Staff at Muhimbili

Birth Registration Interview Guide for Healthcare/Admin Staff at Muhimbili

Study title:

Facility-based birth notification/registration systems analysis in two hospitals in Dar es Salaam, Tanzania

Investigator name:

"Hello, my name is [X] and I will be conducting this interview regarding the topic of facility-based birth notification and registration systems in Tanzania. I want to remind you that all of your responses are confidential, and your name will not be used in my report. The interview should take up to 45 minutes of your time. I will be recoding our interview and will later transcribe the notes. Please feel free to ask me questions throughout the interview, and/or request the interview to be stopped. Thank you again for consenting to meet with me and for providing your time towards my research."

Introduction/Interviewee Information:
Organization:
Name/Title:

Background

1. Please describe your role at the hospital

Birth Notification/Registration/Certification System Barriers/Enablers

"I am now going to ask you a few questions about the birth notification, registration, and certificate systems at your hospital, and your experience with them."

Birth Notification

2. Please describe the birth notification and registration system at Muhimbili.
3. Please describe your responsibilities in relation to the birth notification and registration system (if any)
    - Approximately how much time per week do you dedicate to your duties related to the birth notification system? How do you feel about that?
4. Do you feel birth notification and registration is a priority? Why?
5. What do you think is working well with the birth notification and registration system? Why?

6. What do you think could be improved with the birth notification and registration system? Why?
7. What would you need in order for these improvements to be possible?
8. What would the hospital need in order for these improvements to be possible?

Birth Certificate

9. Please describe the process of getting a birth certificate.
10. Please describe your responsibilities in relation to the birth certificate system (if any)
    - Approximately how much time per week do you dedicate to your duties related to the birth notification system? Why do you feel about that?
11. Do you feel it is important to have a birth certificate? Why?
12. What do you think is working well with the birth certificate system? Why?
13. What do you think could be improved with the birth certificate system? Why?
14. What would you need in order for these improvements to be possible?
15. What would the hospital need in order for these improvements to be possible?

Closing

16. Is there anything more you would like to add about your experiences with, or views on, any of these processes?

"Thank you very much for your time and participation in my research. I will be compiling the information you and other gave me and submitting a report to my organization in one month. If you have any further questions or comments, please feel free to contact me either via email: [X]@student.lshtm.ac.uk or mobile: [X]."

**Themes**	**Questions**
**Birth Notification and Registration**	
Involvement with the birth notification process	Please describe the birth notification and registration system at Muhimbili.<br><br>Please describe your responsibilities in relation to the birth notification and registration system<br><br>Approximately how much time per week do you dedicate to your duties related to the birth notification system? How do you feel about that?
Value for Birth Notification and Registration	Do you feel birth notification and registration is a priority? Why?
Perceived Barriers to Birth Notification	What do you think is working well with the birth notification and registration system? Why?
Perceived Facilitators to Birth Notification	What do you think could be improved with the birth notification and registration system? Why?

What could be done to help improve the process?	What would you need in order for these improvements to be possible?<br><br>What would the hospital need in order for these improvements to be possible?
**Birth Certification**	
Involvement with the birth certification process	Please describe the process of getting a birth certificate.<br><br>Please describe your responsibilities in relation to the birth certificate system (if any)<br><br>Approximately how much time per week do you dedicate to your duties related to the birth notification system? Why do you feel about that?
Value for Birth Certification	Do you feel birth certification and registration is a priority? Why?
Perceived Facilitators	What do you think is working well with the birth certificate system? Why?
Perceived Barriers	What do you think could be improved with the birth certificate system? Why?
What could be done to help improve the process?	What would you need in order for these improvements to be possible?<br><br>What would the hospital need in order for these improvements to be possible?

Mothers at Muhimbili

Birth Notification/Registration/Certification Interview Guide for Mothers in the Maternal Ward at Muhimbili

Study title:

Facility-based birth notification/registration systems analysis in two hospitals in Dar es Salaam, Tanzania

Investigator name:

"Hello, my name is [X] and I will be conducting this interview regarding the topic of facility-based birth notification and registration systems in Tanzania. I want to remind you that all of your responses are confidential, and your name will not be used in my report. The interview should take up to 30 minutes of your time. I will be recoding our interview and will later transcribe the notes. Please feel free to ask me questions throughout the interview, and/or request the interview to be stopped. Thank you again for consenting to meet with me and for providing your time towards my research."

Introduction/interviewee information:
Name:
Number of children:
Age(s) of child/children:
Place(s) of delivery:

Birth Notification

"I am now going to ask you a few questions about the documentation of your child/children's birth/s after they were born in the facility/facilities."(bring example notification card for clarification if the mother is confused by question #1)

1. After delivering your baby, did you receive a birth notification slip?
2. Please describe who gave you the document, and any instructions or information you may have received about the document
3. Was the information you were given clear?
4. Is there anything that could have been done to make the information delivery clearer? (e.g. timing, delivery, document itself, etc.)
5. Do you know what to do with this document?

Birth Certification

"I am now going to ask you a few questions about birth certificates." (bring example of certificate for clarification)

6. Can you please describe how you can get a birth certificate for your child?
7. Do you plan to get a birth certificate for your child? Why?
    - If **yes**:
        1. When do you plan to do this?
        2. Is there anything about the process that might make it difficult for you to get a birth certificate (e.g. time, distance)?

i. If yes, how do you plan to overcome this/these difficulty/difficulties?
3. Do you feel that the process is straightforward and manageable?
4. What parts of the process will make it easier for you to get a birth certificate (e.g. the fact that you can return to your local facility, the fact that it's free, etc.)?

- If **no**:
  1. Is there anything about the process that makes it difficult for you to get a birth certificate (e.g. time, distance)?

8. Is there anything that could be done to improve the process for you, or make it easier?
9. Do you think it's important for your child to have a birth certificate? Why?

If she has other children

10. Did you receive a birth certificate for your other child/children?
    - If **yes**:
      1. How old was your child when you received the birth certificate?
      2. Please tell me about the process of obtaining a birth certificate.
         i. Was the process easy to follow?
         ii. Did you have any difficulties obtaining the birth certificate?
         iii. (If yes) How did you overcome these difficulties?
         iv. Is there anything that would have made the process easier?
         v. Did anyone help you through the process? (If yes, who?)
         vi. Was the process you went through different from the one you were instructed to go through for this most recent baby?
             - If yes: How so? Does this new process sound easier or more difficult?
      3. (if she has multiple children) Did you obtain certificates for all of your children? Were there any differences in your experiences with the processes? (e.g. child care for other children, etc.)
    - If **no**:
      1. Was there anything that made it difficult for you to obtain the birth certificate? (e.g. cost, distance/travel, complicated process, father unavailable, child's name unavailable, etc.)
      2. What could have helped you to get a birth certificate for your child?
      3. (If she has multiple children) did you obtain birth certificates for any of your other children?
      4. Were the instructions for receiving a birth certificate for your previous child/children different from the instructions you received for this most recent baby (if applicable)?
         i. If yes, does this new process sound easier or more difficult?

Closing

11. Is there anything more you would like to add about your experiences with any of these processes? Do you have any further recommendations on how they could have been improved for you?

"Thank you very much for your time and participation in my research. I will be compiling the information you and other gave me and submitting a report to my organization in one month. If you have any further questions or comments, please feel free to contact me either via email: [X]@student.lshtm.ac.uk or mobile: [X]."

Themes	Questions
**Birth Notification**	
Knowledge of the birth notification process	After delivering your baby, did you receive a birth notification card?<br>Did you know what to do with this card or number?<br>(If received) What did you do with this document?<br>Please describe who gave you the document or number, and any instructions or information you may have received about the document or number.
What could be done to help improve the process?	Was the information you were given clear?<br>Is there anything that could have been done to make the process/information delivery clearer? (e.g. timing, delivery, document/number itself, etc.)
**Birth Registration**	
Knowledge of the birth registration process	Can you please describe a birth registration for me?<br>Can you please tell me about any instructions you were given on how to register your child's birth? Who (if anyone) gave you the instructions?<br>Do you think it's important for your child to be registered? Why?
What could be done to help improve the process?	What could help you in registering the birth of your child?<br>Is there anything that would have made the process easier?
**Birth Certification**	

Knowledge of the birth certification process	Can you please describe how you would get a birth certificate for your child?<br>Who or what provided you with this information?<br>Do you think it's important for your child to have a birth certificate? Why?
Perceived barriers	Was there anything that might make it difficult for you to obtain the birth certificate?<br>(If yes), how will you overcome these difficulties?
Perceived enablers	Is the process easy to follow?<br>Was there anyone that helped you with the process? Who?
What could be done to help improve the process?	What could help you in getting a birth certificate for your child?<br>Is there anything that would make the process easier?

Staff at Temeke

Birth Registration Interview Guide for Healthcare/Admin Staff at Temeke

Study title:

Facility-based birth notification/registration systems analysis in two hospitals in Dar es Salaam, Tanzania

Investigator name:

"Hello, my name is [X] and I will be conducting this interview regarding the topic of facility-based birth registration and certification systems in Tanzania. I want to remind you that all of your responses are confidential, and your name will not be used in my report. The interview should take up to 45 minutes of your time. I will be recoding our interview and will later transcribe the notes. Please feel free to ask me questions throughout the interview, and/or request the interview to be stopped. Thank you again for consenting to meet with me and for providing your time towards my research."

Introduction/Interviewee Information:
Organization:
Name/Title:

Background

1. Please describe your role at the hospital

Birth Registration/Certification System Barriers/Enablers

"I am now going to ask you a few questions about the birth registration and certification systems at your hospital, and your experience with them."

Birth Registration

2. Please describe the birth registration system at Temeke.
3. Please describe your responsibilities in relation to the birth registration system (if any)
    - Approximately how much time per week do you dedicate to your duties related to the birth registration system? How do you feel about that?
4. Do you feel birth registration is a priority? Why?
5. What do you think is working well with the birth notification and registration system? Why?
6. What do you think could be improved with the birth registration system? Why?
7. What would you need in order for these improvements to be possible?
8. What would the hospital need in order for these improvements to be possible?

Birth Certificate

9. Please describe the process of getting a birth certificate at Temeke.
10. Please describe your responsibilities in relation to the birth certificate system (if any) (individual responses)
    - Approximately how much time per week do you dedicate to your duties related to the birth notification system? Why do you feel about that?
11. Do you feel it is important to have a birth certificate? Why?

12. What do you think is working well with the birth certificate system? Why?
13. What do you think could be improved with the birth certificate system? Why?
14. What would you need in order for these improvements to be possible?
15. What would the hospital need in order for these improvements to be possible?

Closing

16. Is there anything more you would like to add about your experiences with, or views on, any of these processes?

"Thank you very much for your time and participation in my research. I will be compiling the information you and other gave me and submitting a report to my organization in one month. If you have any further questions or comments, please feel free to contact me either via email: [X]@student.lshtm.ac.uk or mobile: [X]."

Themes	Questions
**Birth Registration**	
Involvement with the birth registration process	Please describe the birth registration system at Temeke.<br>Please describe your responsibilities in relation to the birth registration system<br>Approximately how much time per week do you dedicate to your duties related to the birth registration system? How do you feel about that?
Value for Birth Registration	Do you feel birth registration is a priority? Why?
Perceived Barriers to Birth Registration	What do you think is working well with the birth registration system? Why?
Perceived Facilitators to Birth Registration	What do you think could be improved with the birth registration system? Why?
What could be done to help improve the process?	What would you need in order for these improvements to be possible?<br>What would the hospital need in order for these improvements to be possible?
**Birth Certification**	
Involvement with the birth certification process	Please describe the process of getting a birth certificate.

	Please describe your responsibilities in relation to the birth certificate system (if any)<br><br>Approximately how much time per week do you dedicate to your duties related to the birth notification system? Why do you feel about that?
Value for Birth Certification	Do you feel birth certification and registration is a priority? Why?
Perceived Facilitators	What do you think is working well with the birth certificate system? Why?
Perceived Barriers	What do you think could be improved with the birth certificate system? Why?
What could be done to help improve the process?	What would you need in order for these improvements to be possible?<br><br>What would the hospital need in order for these improvements to be possible?

Mothers in the Maternal Ward at Temeke

Birth Registration/Certification Interview Guide for Mothers in the Registration Area at Temeke

Study title:

Facility-based birth registration systems analysis in two hospitals in Dar es Salaam, Tanzania

Investigator name:

"Hello, my name is [X] and I will be conducting this interview regarding the topic of facility-based birth registration system in Tanzania. I want to remind you that all of your responses are confidential, and your name will not be used in my report. The interview should take up to 30 minutes of your time. I will be recoding our interview and will later transcribe the notes. Please feel free to ask me questions throughout the interview, and/or request the interview to be stopped. Thank you again for consenting to meet with me and for providing your time towards my research."

Introduction/interviewee information:

Name:
Number of children:
Age(s) of child/children:
Place(s) of delivery:

Birth Registration

"I am now going to ask you a few questions about the documentation of your child/children's birth/s after they were born in the facility/facilities."

1. After delivering your baby, did you receive a delivery card?
2. Please describe who gave you the document, and any instructions or information you may have received about the document
3. Was the information you were given clear?
4. Is there anything that could have been done to make the information delivery clearer? (e.g. timing, delivery, document itself, etc.)

Birth Certification

"I am now going to ask you a few questions about birth certificates." (bring example of certificate for clarification)

1. Please tell me about the process of obtaining a birth certificate.
    1. Was the process easy to follow?
    2. Did you have any difficulties with the process (e.g. distance/travel, complicated instructions, keeping track of the birth notification slip, childcare for other children, etc.)?
    3. (If yes) How did you overcome these difficulties?
    4. Is there anything that would have made the process easier?
    5. Did anyone help you through the process? (If yes, who?)

2. (If she has multiple children) Did you obtain certificates for all of your children? Were there any differences in your experiences with the processes?
3. Do you think it's important for your child to have a birth certificate? Why?

Closing

4. Is there anything more you would like to add about your experiences with any of these processes? Do you have any further recommendations on how they could have been improved for you?

"Thank you very much for your time and participation in my research. I will be compiling the information you and other gave me and submitting a report to my organization in one month. If you have any further questions or comments, please feel free to contact me either via email: [X]@student.lshtm.ac.uk or mobile: [X]."

Themes	Questions
**Birth Registration**	
Knowledge of the birth registration process	After delivering your baby, did you receive a delivery card?<br><br>Please describe who gave you the document, and any instructions or information you may have received about the document
What could be done to help improve the process?	Was the information you were given clear?<br><br>Is there anything that could have been done to make the information delivery clearer? (e.g. timing, delivery, document itself, etc.)
**Birth Certification**	
Knowledge of the birth certification process	Please tell me about the process of obtaining a birth certificate.<br><br>(If she has multiple children) Did you obtain certificates for all of your children? Were there any differences in your experiences with the processes?<br><br>Do you think it's important for your child to have a birth certificate? Why?
Perceived barriers	Did you have any difficulties with the process (e.g. distance/travel, complicated instructions,

	keeping track of the birth notification slip, childcare for other children, etc.)?<br><br>(If yes) How did you overcome these difficulties?
Perceived enablers	Was the process easy to follow?<br><br>Is there anything that would have made the process easier?<br><br>Did anyone help you through the process? (If yes, who?)
What could be done to help improve the process?	What could have helped you in getting a birth certificate for your child?<br><br>Is there anything that would have made the process easier?

Mothers in the Registration Area at Temeke

Birth Registration/Certification Interview Guide for Mothers in the Registration Area at Temeke

Study title:

Facility-based birth registration systems analysis in two hospitals in Dar es Salaam, Tanzania

Investigator name:

"Hello, my name is [X] and I will be conducting this interview regarding the topic of facility-based birth registration system in Tanzania. I want to remind you that all of your responses are confidential, and your name will not be used in my report. The interview should take up to 30 minutes of your time. I will be recoding our interview and will later transcribe the notes. Please feel free to ask me questions throughout the interview, and/or request the interview to be stopped. Thank you again for consenting to meet with me and for providing your time towards my research."

Introduction/interviewee information:

Name:
Number of children:
Age(s) of child/children:
Place(s) of delivery:

Birth Registration

"I am now going to ask you a few questions about the documentation of your child/children's birth/s after they were born in the facility/facilities."

5. After delivering your baby, did you receive a delivery card?
6. Please describe who gave you the document, and any instructions or information you may have received about the document
7. Was the information you were given clear?
8. Is there anything that could have been done to make the information delivery clearer? (e.g. timing, delivery, document itself, etc.)

Birth Certification

"I am now going to ask you a few questions about birth certificates." (bring example of certificate for clarification)

5. Please tell me about the process of obtaining a birth certificate.
    1. Was the process easy to follow?
    2. Did you have any difficulties with the process (e.g. distance/travel, complicated instructions, keeping track of the birth notification slip, childcare for other children, etc.)?
    3. (If yes) How did you overcome these difficulties?
    4. Is there anything that would have made the process easier?
    5. Did anyone help you through the process? (If yes, who?)

6. (If she has multiple children) Did you obtain certificates for all of your children? Were there any differences in your experiences with the processes?
7. Do you think it's important for your child to have a birth certificate? Why?

Closing

8. Is there anything more you would like to add about your experiences with any of these processes? Do you have any further recommendations on how they could have been improved for you?

"Thank you very much for your time and participation in my research. I will be compiling the information you and other gave me and submitting a report to my organization in one month. If you have any further questions or comments, please feel free to contact me either via email: [X]@student.lshtm.ac.uk or mobile: [X]."

Themes	Questions
**Birth Registration**	
Knowledge of the birth registration process	After delivering your baby, did you receive a delivery card?<br><br>Please describe who gave you the document, and any instructions or information you may have received about the document
What could be done to help improve the process?	Was the information you were given clear?<br><br>Is there anything that could have been done to make the information delivery clearer? (e.g. timing, delivery, document itself, etc.)
**Birth Certification**	
Knowledge of the birth certification process	Please tell me about the process of obtaining a birth certificate.<br><br>(If she has multiple children) Did you obtain certificates for all of your children? Were there any differences in your experiences with the processes?<br><br>Do you think it's important for your child to have a birth certificate? Why?
Perceived barriers	Did you have any difficulties with the process (e.g. distance/travel, complicated instructions,

	keeping track of the birth notification slip, childcare for other children, etc.)?<br><br>(If yes) How did you overcome these difficulties?
Perceived enablers	Was the process easy to follow?<br><br>Is there anything that would have made the process easier?<br><br>Did anyone help you through the process? (If yes, who?)
What could be done to help improve the process?	What could have helped you in getting a birth certificate for your child?<br><br>Is there anything that would have made the process easier?

Stakeholders

Birth Registration/Certification and CRVS Interview Guide for Key Stakeholders

Study title:

Facility-based birth notification/registration systems analysis in two hospitals in Dar es Salaam, Tanzania

Investigator name:

"Hello, my name is [X] and I will be conducting this interview regarding the topic of facility-based birth notification and registration systems in Tanzania. I want to remind you that all of your responses are confidential, and your name will not be used in my report. The interview should take up to 30 minutes of your time. I will be recoding our interview and will later transcribe the notes. Please feel free to ask me questions throughout the interview, and/or request the interview to be stopped. Thank you again for consenting to meet with me and for providing your time towards my research."

Introduction/Interviewee Information:

Name:
Organization:
Title:

Background

1. Please describe your role.

Birth Notification/Registration/Certificate Systems

"I am now going to ask you a few questions about the birth notification, registration, certificate, and CRVS systems in Tanzania, and your experience with them."

2. Please describe Tanzania's birth notification and registration system.
3. What are your responsibilities with the birth notification/registration/certificate systems? Please describe.
4. Do you feel birth notification and registration is a national priority? Why?
5. What do you think is working well with the birth notification and registration system? Why?
6. What do you think could be improved with the birth notification and registration system? Why?
7. Do you foresee any challenges in implementing these improvements? Please describe.
8. Who would need to be involved to make these improvements happen?

Birth Certificate

9. Do you feel it is a national priority for Tanzanian citizens to possess a birth certificate? Why?
10. What do you think is working well with the birth certificate system? Why?
11. What do you think could be improved with the birth certificate system? Why?
12. Do you foresee any challenges in implementing these improvements? Please describe.
13. Who would need to be involved to make these improvements happen?

CRVS System

14. Please describe Tanzania's CRVS system
15. What is your involvement with the wider CRVS system? Please describe.
16. What do you think is working well with the CRVS system? Why?
17. What do you think could be improved with the CRVS system? Why?
18. Do you foresee any challenges in implementing these improvements? Please describe.
19. Who would need to be involved to make these improvements happen?

Closing

20. Is there anything more you would like to add about your experiences with, or views on, any of these processes?

"Thank you very much for your time and participation in my research. I will be compiling the information you and other gave me and submitting a report to my organization in one month. If you have any further questions or comments, please feel free to contact me either via email: [X]@student.lshtm.ac.uk or mobile: [X]."

**Themes**	**Questions**
**Birth Registration**	
Involvement with the birth registration process	Please describe Tanzania's birth notification and registration system.<br>What are your responsibilities with the birth notification/registration/certificate systems? Please describe.
Value for Birth Registration	Do you feel birth registration is a priority? Why?
Perceived Facilitators to Birth Registration	What do you think is working well with the birth registration system? Why?
Perceived Barriers to Birth Registration	What do you think could be improved with the birth registration system? Why?
What could be done to help improve the process?	Do you foresee any challenges in implementing these improvements? Please describe.<br>Who would need to be involved to make these improvements happen?
**Birth Certification**	

Value for Birth Certification	Do you feel it is a national priority for Tanzanian citizens to possess a birth certificate? Why?
Perceived Facilitators	What do you think is working well with the birth certificate system? Why?
Perceived Barriers	What do you think could be improved with the birth certificate system? Why?
What could be done to help improve the process?	Do you foresee any challenges in implementing these improvements? Please describe.<br>Who would need to be involved to make these improvements happen?
**CRVS**	
Involvement with the CRVS system	Please describe Tanzania's CRVS system<br>What is your involvement with the wider CRVS system? Please describe.
Perceived Facilitators	What do you think is working well with the CRVS system? Why?
Perceived Barriers	What do you think could be improved with the CRVS system? Why?
What could be done to help improve the process?	Do you foresee any challenges in implementing these improvements? Please describe.<br>Who would need to be involved to make these improvements happen?
