# Supplementary material for: Counting on birth registration: mixed-methods research in two EN-BIRTH study hospitals in Tanzania
Source: BMC Pregnancy Childbirth. 2021 Mar 26;21(Suppl 1):236. doi: 10.1186/s12884-020-03357-1 (PMC7995691; doi:10.1186/s12884-020-03357-1)
Supplement: Supplementary file 4 — Additional file 4. Birth registration thematic content analysis, EN-BIRTH study. [file 12884_2020_3357_MOESM4_ESM.pdf]

Every Newborn BIRTH multi-country validation study: informing measurement of coverage and quality of maternal and newborn care

Counting on birth registration: mixed-methods research in two EN-BIRTH study hospitals in Tanzania

Additional File 4: Birth registration thematic content analysis, EN-BIRTH study

Temeke Hospital

No.	Title/ID	Ward/Office	Barriers	Enablers	Recommendations
1	Mother 1, Temeke	Registration	• **Incomplete, inconsistent, and/or misunderstood instructions**: was unaware that she had to come with both cards so was sent back the first time, was under the impression that the hand-written certificates were only valid for 5 years after which she would have to go to the District Office for a printed certificate<br>• **Distance/cost of transport**	• **Understood importance of birth certificate** (cited education)<br>• **Certificate provided for free**	• Need for clear instructions<br>• Provide registration services at all hospitals where deliveries occur
2	Mother 2, Temeke	Registration	• **Incomplete, inconsistent, and/or misunderstood instructions**: cited 45 days instead of 42	• **Understood importance of birth certificate** (cited education, employment, travel)<br>• **Reduced distance** (she lived near Temeke)	• Continue with the UNICEF process (went through the national process with prior children and preferred this one)
3	Mother 3, Temeke	Postnatal	• **Shortage of resources**: mother had to return to Temeke 1 week after discharge to receive the delivery card<br>• **Incomplete, inconsistent, and/or misunderstood instructions**: cited 43 days instead of 42<br>• **Amount of time** between delivery and certification	• **Understood importance of birth certificate** (all four of her children have one)	• Reduce the amount of time between delivery and picking up a certificate
4	Mother 4, Temeke	Postnatal	• **Distance**	• **Understood importance of birth certificate**	• Certificate should be provided prior to

				(cited that it gives the baby an identity, records date of birth, employment, school)	discharge at the facility
5	Mother 5, Temeke	Registration	• **Incomplete, inconsistent, and/or misunderstood instructions**: cited 40 days instead of 42<br>• **Shortage of space/resources**: didn't know where to go once she got to Temeke because there is no designated space	• **Understood importance of birth certificate** (cited education, travel)	• **Certificates should be provided in every hospital where deliveries occur**<br>• Certificates (under the UNICEF program) should be provided for children over 5 years of age
6	Mother 6, Temeke	Postnatal	• **Incomplete, inconsistent, and/or misunderstood instructions**: stated that there was no clear or accurate information provided<br>• **Time** required between delivery and certification	• **Understood importance of birth certificate** (cited education, travel, employment)	• They [the nurses] should provide clear information<br>• Certificates should be provided without delays
7	Health Worker 1, Temeke	Registration	• **Incomplete, inconsistent, and/or misunderstood instructions**: stated that certificates are provided immediately after delivery, and also said they advise mothers to wait 42 days after delivery<br>• **Shortage of space**: there is no formal office or area for getting a birth certificate, and the healthcare provider responsible has no space to store her documents<br>• **Lack of motivation and training for staff:** she is trained as a healthcare provider but has been assigned to write birth certificates	• **Certificate provided free of charge**<br>• **Increased awareness of importance of certification**<br>• **Reduced time**: certificates are provided same-day so that mothers don't "lose hope"	• **Certificates should be provided in every hospital where deliveries occur**<br>• All hospitals should adopt the UNICEF process<br>• **Provide trainings for staff**<br>• Provide space for registration
8	Health Worker 2, Temeke	Postnatal	• **Shortage of staff:** need additional nurses to be filling out delivery cards	• **Increased awareness** (among mothers)	• Assign a designated person to fill out delivery cards

			while others are tending to deliveries<br>• **Shortage of space/resources**: mothers are supposed to be kept in the facility 24 hours after delivery, but due to a shortage of beds the are only kept for 6, also not enough delivery and clinic cards<br>• **Lack of training for staff**	of the process and importance	• **Provide training for staff**
9	Health Worker 3, Temeke	Postnatal	• **Shortage of resources**: don't always have enough delivery cards for mothers<br>• **Shortage of staff**<br>• **Lack of motivation and training for staff**<br>• **Time:** not possible for mother to receive birth certificate before 42 days because the baby won't have a name yet	• New procedure is easier for mothers because they no longer have to keep track of birth notification cards	• **Provide training for staff**<br>• Increased staff<br>• Increased materials (delivery cards)
10	Health Worker 4, Temeke	HMIS	• **Shortage of staff** to provide birth certificates (though she recommended using technology instead of hiring new staff)	• **Increased awareness** (among mothers) of the importance of certification<br>• Laws that mandate a birth certificate for access to services (e.g. education)<br>• Decreased distance for most as opposed to going to the District Office	• Electronic registration system to decrease time of writing certificates and improve quality

Muhimbili Hospital

No.	Title	Ward/Office	Barriers	Enablers	Recommendations
11	Mother 7, Muhimbili	Postnatal	• **Incomplete, inconsistent, and/or misunderstood instructions:** didn't	-	-

			understand or recall instructions given<br>• Was not aware of importance of birth certificate (said maybe she was distracted or missed the instructions and explanation)<br>• **Time** "they didn't provide the certificates at the right time"<br>• **Distance** to travel to the District Office		
14	Mother 8, Muhimbili	KMC	• **Incomplete, inconsistent, and/or misunderstood instructions:** didn't understand or recall instructions, such as where to go to get the birth certificate	• **Understood importance of birth certificate** (cited access to education)	• Provide clear information and instructions
15	Mother 9, Muhimbili	KMC	• **Incomplete, inconsistent, and/or misunderstood instructions:** didn't recall instructions<br>• **Concerns with keeping track of birth notification card**<br>• **Distance**	• **Understood importance of birth certificate** (cited access to education, employment, recognition of date of birth)	-
16	Mother 10, Muhimbili	KMC	• **Distance**<br>• **Lack of time to travel and complete the process**	• **Understood importance of birth certificate** (cited access to education and employment)	• "They should provide the birth certificates on time"
12	Health Worker 5, Muhimbili	HMIS	• **Perceived lack of value and understanding by mothers**<br>• **Lack of electronic system for information storage**	• Expressed importance of birth registration (but only reason given was that it was good data for RITA)	• Government should promote importance of birth registration./certification via media<br>• Electronic system for record storage
13	Health Worker 6, Muhimbili	HMIS	• **Lack of electronic system for information storage**<br>• Acknowledged issues with distance and cost for mothers	• Expressed value for birth registration as a hospital	• Expand hospital's digital system to include birth registration records (already have a system, electricity, and internet at the hospital)

17	Health Worker 7, Muhimbili	Postnatal	• **Perceived lack of value and understanding by mothers**	• Strong system in place to ensure every mother leaves the facility with a birth notification<br>• Laws that mandate a birth certificate for access to services (e.g. education)	• More/improved education for mothers on the importance and process of birth registration/certification

Stakeholders

No.	Title	Ward/Office	Barriers	Enablers	Recommendations
18	Stakeholder 1	UNICEF	• Current system is demand-unfriendly,<br>• Expensive (for demand-side)<br>• Involves multiple visits<br>• RITA sees birth certification to be an income-generating service	• Mothers are responsible and "99% have either of the two documents" required for certification	• One-step, one-visit<br>• Reduce costs<br>• Increase awareness<br>• Bring registration and certification services to the ward level
19	Stakeholder 2	RITA	• Financial constraints<br>• Lack of electricity nation-wide<br>• Lack of offices/space to keep vital records (because most aren't digitized)	• Marketing manager in place who produces leaflets, TV programs, news, radio, etc, to spread awareness<br>• UNICEF under 5 registration initiative showing great success<br>• Reported to allow flexibility with the under 5 program (in terms of timing and documentation)	• Need to make registration jobs and duties into law
20	Stakeholder 3	MoH	• Cost (for families to get birth certificates)<br>• Poor accessibility<br>• Lack of awareness<br>• Shortage of funds	• Strategy work already underway by government and other key stakeholders	• Law must be adopted to formalize initiative

21	Stakeholder 4	MoH	• National-level awareness<br>• Lack of synergy between all governmental and partnering stakeholders<br>• Cost (for families to get birth certificate)	• CRVS Strategy has been created<br>• Awareness is improving and it's become a government priority	-
