# Supplementary material for: Counting on birth registration: mixed-methods research in two EN-BIRTH study hospitals in Tanzania
Source: BMC Pregnancy Childbirth. 2021 Mar 26;21(Suppl 1):236. doi: 10.1186/s12884-020-03357-1 (PMC7995691; doi:10.1186/s12884-020-03357-1)
Supplement: Supplementary file 5 — Additional file 5. Exit survey-reported birth notification and certificate coverage, stratified by education level and socioeconomic status. [file 12884_2020_3357_MOESM5_ESM.pdf]

Every Newborn BIRTH multi-country validation study: informing measurement of coverage and quality of maternal and newborn care

Counting on birth registration: mixed-methods research in two EN-BIRTH study hospitals in Tanzania

Additional File 5: Exit survey-reported birth notification and certificate coverage, stratified by education level and socioeconomic status

	Reports received	*Reports not received*	*Don't know*
**Birth Notification - Education Level**			
**Muhimbili**			
No education	46 (1.9)	1 (1.1)	0 (0)
Primary incomplete	39 (1.6)	1 (1.1)	0 (0)
Primary complete	798 (33.2)	36 (39.1)	0 (0)
Secondary incomplete	769 (31.9)	20 (21.7)	0 (0)
Secondary complete	748 (31.1)	34 (37)	1 (100)
Missing	7 (0.3)	0 (0)	0 (0)
Total	2407 (100)	92 (100)	1 (100)
**Temeke**			
No education	49 (2.7)	98 (2.9)	16 (4.4)
Primary incomplete	28 (1.5)	69 (2.1)	9 (2.5)
Primary complete	1061 (58)	1911 (57.1)	206 (56.9)
Secondary incomplete	391 (21.4)	755 (22.6)	96 (26.5)
Secondary complete	295 (16.1)	503 (15)	33 (9.1)
Missing	4 (0.2)	12 (0.4)	2 (0.6)
Total	1828 (100)	3348 (100)	362 (100)
**Birth Notification - Wealth quintile**			
**Muhimbili**			
Lowest	430 (17.9)	29 (31.5)	0 (0)
Second	459 (19.1)	15 (16.3)	0 (0)
Middle	498 (20.7)	15 (16.3)	1 (100)
Fourth	471 (19.6)	17 (18.5)	0 (0)
Highest	527 (21.9)	14 (15.2)	0 (0)
Total	2407 (100)	92 (100)	1 (100)
**Temeke**			
Lowest	348 (19)	626 (18.7)	118 (32.6)
Second	309 (16.9)	791 (23.6)	95 (26.2)
Middle	560 (30.6)	605 (18.1)	59 (16.3)
Fourth	233 (12.7)	607 (18.1)	40 (11)
Highest	360 (19.7)	693 (20.7)	48 (13.3)
Missing	18 (1)	26 (0.8)	2 (0.6)

	Reports received	*Reports not received*	*Don't know*
Total	1828 (100)	3348 (100)	362 (100)
**Birth Certification – Education level**			
**Muhimbili**			
No education	23 (2)	23 (1.7)	1 (4.2)
Primary incomplete	19 (1.7)	21 (1.6)	0 (0)
Primary complete	396 (35.2)	427 (31.6)	11 (45.8)
Secondary incomplete	352 (31.3)	433 (32.1)	4 (16.7)
Secondary complete	331 (29.4)	444 (32.9)	8 (33.3)
Missing	4 (0.4)	3 (0.2)	0 (0)
Total	1125 (100)	1351 (100)	24 (100)
**Temeke**			
No education	4 (1.5)	159 (3.1)	0 (0)
Primary incomplete	7 (2.6)	98 (1.9)	1 (2)
Primary complete	158 (57.7)	2989 (57.3)	31 (60.8)
Secondary incomplete	57 (20.8)	1175 (22.5)	10 (19.6)
Secondary complete	48 (17.5)	775 (14.9)	8 (15.7)
Missing	0 (0)	17 (0.3)	1 (2)
Total	274 (100)	5213 (100)	51 (100)
**Birth Certification – Wealth quintiles**			
**Muhimbili**			
Lowest	212 (18.8)	239 (17.7)	8 (33.3)
Second	226 (20.1)	243 (18)	5 (20.8)
Middle	266 (23.6)	245 (18.1)	3 (12.5)
Fourth	237 (21.1)	247 (18.3)	4 (16.7)
Highest	171 (15.2)	366 (27.1)	4 (16.7)
Missing	13 (1.2)	11 (0.8)	0 (0)
Total	1125 (100)	1351 (100)	100)
**Temeke**			
Lowest	44 (16.1)	1034 (19.8)	14 (27.5)
Second	24 (8.8)	1160 (22.3)	11 (21.6)
Middle	106 (38.7)	1109 (21.3)	9 (17.6)
Fourth	32 (11.7)	840 (16.1)	8 (15.7)
Highest	67 (24.5)	1025 (19.7)	9 (17.6)
Missing	1 (0.4)	45 (0.9)	0 (0)
Total	274 (100)	5213 (100)	51 (100)
