# Supplementary material for: Counting on birth registration: mixed-methods research in two EN-BIRTH study hospitals in Tanzania
Source: BMC Pregnancy Childbirth. 2021 Mar 26;21(Suppl 1):236. doi: 10.1186/s12884-020-03357-1 (PMC7995691; doi:10.1186/s12884-020-03357-1)
Supplement: Supplementary file 6 — Additional file 6. Ethical approval of local institutional review boards, EN-BIRTH study. [file 12884_2020_3357_MOESM6_ESM.pdf]

Every Newborn BIRTH multi-country validation study: informing measurement of coverage and quality of maternal and newborn care

Counting on birth registration: mixed-methods research in two EN-BIRTH study hospitals in Tanzania

Additional File 6: Ethical approval of local institutional review boards, EN-BIRTH study

Country	Institutional Review Boards	Date	Number/Ref
UK	London School of Hygiene & Tropical Medicine (LSHTM) Interventions Research Ethics Committee	03.10.16	13808 and 11780
Tanzania	National Institute for Medical Research (NIMRI)	20.01.17	NIMR/HQ/R.8a/Vol IX/2394
	Ifakara Health Institute	20.10.16	IHI/IRB/No: 032-2016
	Muhimbili University of Health and Allied Sciences research and Publications committee	21.10.16	2016-10-21- /AEC/Vol.XI/310
